# Drug-Induced Immune Hemolytic Anemia due to Amoxicillin-Clavulanate: A Case Report and Review

**DOI:** 10.7759/cureus.8666

**Published:** 2020-06-17

**Authors:** Janet Chan Gomez, Tabinda Saleem, Samantha Snyder, Maria Joseph, Tejaswi Kanderi

**Affiliations:** 1 Internal Medicine, University of Pittsburgh Medical Center (UPMC) Pinnacle, Harrisburg, USA

**Keywords:** amoxicillin-clavulanate, anemia, hemolytic anemia, hematology, drug-induced immune hemolytic anemia

## Abstract

Drug-induced immune hemolytic anemia (DIIHA) is a rare cause of anemia. It is often difficult to distinguish from other causes of hemolytic anemia, thereby delaying diagnosis and treatment. Antibiotics, including penicillins and cephalosporins, are the drugs most often implicated in the development of DIIHA. Discontinuation of the offending agent is often sufficient for treatment. Here, we review the case of a 25-year-old Caucasian female who presented with jaundice and generalized weakness in the setting of outpatient treatment with amoxicillin-clavulanate due to sinus infection. Laboratory testing revealed transaminitis and hemolytic anemia. Direct antiglobulin test (DAT) revealed negative IgG and positive anti-C3. Cold agglutinin titer and Donath-Landsteiner test were negative. The patient was diagnosed with DIIHA most likely due to amoxicillin. She improved with drug cessation and a short course of glucocorticoids. Mechanism of DIIHA, workup, and management are subsequently reviewed.

## Introduction

Drug-induced immune hemolytic anemia (DIIHA) is a rare cause of anemia with an estimated incidence of one to two cases per million worldwide [1-3]. If not recognized early, DIIHA can have deleterious complications such as massive hemolysis that can lead to shock, organ ischemia, disseminated intravascular coagulation (DIC), and acute respiratory distress syndrome (ARDS) [3]. Antibiotics are the most often implicated agents in the development of DIIHA, with the penicillin and cephalosporin classes being the most common in this group [1,4-6]. Amoxicillin-clavulanate is a commonly used antibiotic in clinical practice and while known to cause DIIHA, few cases have been reported in the literature.

## Case presentation

A 25-year-old Caucasian female with a history of major depression with psychotic features presented with new-onset generalized weakness, dark urine, and jaundice. The patient noted on admission that she was on day 5 out of seven days of amoxicillin-clavulanate for the treatment of a sinus infection, the symptoms of which had completely resolved. On admission, vital signs were unremarkable. Physical exam was significant for jaundice with scleral icterus, but no hepatomegaly or splenomegaly was present.

Laboratory testing revealed anemia with low hemoglobin (Hb) and hematocrit (Hct) level (patient's baseline Hb 14). Liver function tests (LFTs) and lactate dehydrogenase (LDH) were both elevated, and haptoglobin was low suggesting a hemolytic anemia (Table 1). A peripheral blood smear (PBS) was completed which revealed spherocytes and degmacytes placing a hemolytic anemia higher on the differential. 

**Table 1:** Outline of patients initial laboratory results

Lab	Value	Reference Range
Hemoglobin (g/dL)	6.8	11.7-15.1
Hematocrit (%)	19.2	29.4-47
Reticulocytes (%)	2.91	0.5-2.17
Haptoglobin (mg/dL)	<8	43-212
Aspartate Aminotransferase (U/L)	133	13-39
Alanine Transaminase (U/L)	187	7-52
Direct Bilirubin (mg/dL)	4.1	0-0.2
Total Bilirubin	8.7	0.3-1
Alkaline Phosphatase (U/L)	163	34-104
Lactate Dehydrogenase (U/L)	891	100-190

The patient reported no personal or family history of anemia or liver disease. She reported consumption of approximately two beers per week and denied use of recreational drugs. She denied any allergies. The patient's home medications included quetiapine, hydroxyzine, and escitalopram. She had been on all of her home medications for over one year with no reported side effects. Due to reported liver dysfunction with quetiapine, this medication was initially held pending further workup.

Ultrasound of the liver was completed and revealed no abnormalities. Antinuclear antibody, hepatitis panel, and urine hemosiderin were negative. Direct antiglobulin test (DAT) revealed negative IgG and positive anti-C3. Cold agglutinin titer and the Donath-Landsteiner test were negative. The patient was subsequently diagnosed with DIIHA secondary to amoxicillin. The patient's Hb continued to trend down, ranging from 5.7 to 7.5 g/dL. She required several transfusions of packed red blood cells (RBCs) to keep an Hb goal of more than 7 g/dL. Due to continued anemia, she was started on a two-week course of prednisone. After four to five days of treatment, her anemia and LFTs improved. She was restarted on quetiapine with no worsening of symptoms. Laboratory tests one week after discharge revealed near normal Hb and LFTs. 

## Discussion

DIIHA is a rare cause of anemia which can present within hours to months after initial drug exposure [4,5,7]. Those who develop hemolysis more quickly typically have a history of exposure to that drug or a drug in the same class [5]. At this time more than 130 drugs are known to cause DIIHA; however, as new drugs are discovered, this number continues to grow [1,2]. The drug classes most commonly implicated are antimicrobials, followed by anti-inflammatory and platinum-based antineoplastic agents [1]. Among the antimicrobials, the most frequent culprits are cephalosporins (with second and third generation being most common) and penicillins [1,3,5,7]. 

Development of DIIHA is primarily due to antibody development [3,8]. Drug-induced antibodies are further classified as drug-dependent antibodies or drug-independent autoantibodies [1-3,9]. Drug-dependent antibodies require the presence of the inciting drug to bind and lyse cells and are the most frequently observed antibodies in DIIHA [1,3,8]. A drug-dependent antibody response is observed in both penicillin- and cephalosporin-induced hemolysis [1-3,8].

In drug-dependent DIIHA, the final immune response is determined by the bond formed between the drug and the RBC membrane [3,7]. A covalent bond results in what is known as the drug absorption process and is the primary response seen with penicillins [3,8]. In this scenario, IgG targets the drug bound to the RBC membrane and macrophages interact resulting in Fc-mediated extravascular hemolysis [1,5]. In ceftriaxone-induced hemolysis, a loose bond between the drug and the RBC membrane results in immune complex formation with complement system activation and intravascular hemolysis [1,3,5,8]. The latter mechanism typically results in worse outcomes and is associated with higher rates of renal failure, DIC, and death [1,3,7].

A less common mechanism in the development of DIIHA involves drug-independent autoantibodies [1,3]. These autoantibodies can be found in DIIHA due to beta-lactamase inhibitors and platinum-based chemotherapeutics [6]. Drug-independent autoantibodies may react and bind to the RBC membrane in the absence of the inciting drug [1-3,8]. These drug-independent autoantibodies are serologically indistinguishable from autoantibodies seen in warm autoimmune hemolytic anemia (WAIHA) [3,9]. When these autoantibodies are the primary mediators of the hemolytic anemia, differentiating between DIIHA and WAIHA relies on improvement of the clinical picture with cessation of the offending drug [1,3]. In some cases, both drug-dependent antibodies and drug-independent autoantibodies are formed resulting in what is known as mixed type DIIHA [1,2] 

Initial symptoms of DIIHA are often vague and are primarily related to the underlying anemia. Symptoms include fatigue, dizziness, dyspnea, jaundice, and dark or bloody urine as were seen in our patient [8,10]. Physical exam may reveal pallor, jaundice, hepatomegaly, splenomegaly, or adenopathy. As drugs which are seemingly benign (such as non-steroidal anti-inflammatory drugs) can be causative agents, a thorough medical and drug history is crucial to the initial workup in all patients with hemolytic anemia [2,4,5].

As in all hemolytic anemias, laboratory workup will reveal a significant drop in Hb and Hct from baseline and mild leukocytosis can be seen [8,10-12]. Early stages may show reduced reticulocyte count [3,11]. Later stages show appropriate reflex increase in reticulocyte counts resulting in elevated mean corpuscular volume (MCV). LDH and indirect bilirubin will be elevated with a reduction in haptoglobin [8,11]. Elevation in LFTs may additionally be seen. A PBS is helpful in the initial diagnosis and may reveal poikilocytosis, schistocytes, spherocytes, anisocytosis, or polychromasia [8,11]. The patient in this case presented with elevated LDH, indirect bilirubin, and LFTs, as well as low haptoglobin. PBS showed spherocytes, which is one of the most common findings in hemolytic anemia.

To confirm diagnosis of DIIHA, DAT is recommended and determines if IgG and/or C3 is bound to the RBC membrane [4-7]. A positive DAT can determine if a hemolytic anemia is due to an immune or nonimmune-mediated etiology [7,12]. Of note, DAT can be positive in a number of other disease states, including: malignancy, post-transfusion/immunoglobulin administration, liver disease, and renal disease [12]. If IgG alone or IgG plus C3 are positive, WAIHA and DIIHA must both be considered and further elution should be completed to determine if an autoantibody is present [4-6]. While the presence of an autoantibody is more suggestive of WAIHA, as previously stated, it does not completely rule out DIIHA due to drug-independent autoantibodies [1,4,8]. It is important to note that WAIHA is more common than DIIHA [5,12]. In this scenario, careful history can help delineate the cause. If any potential offending agents are found, they should can be discontinued [1,4,6,7]. 

**Figure 1:**
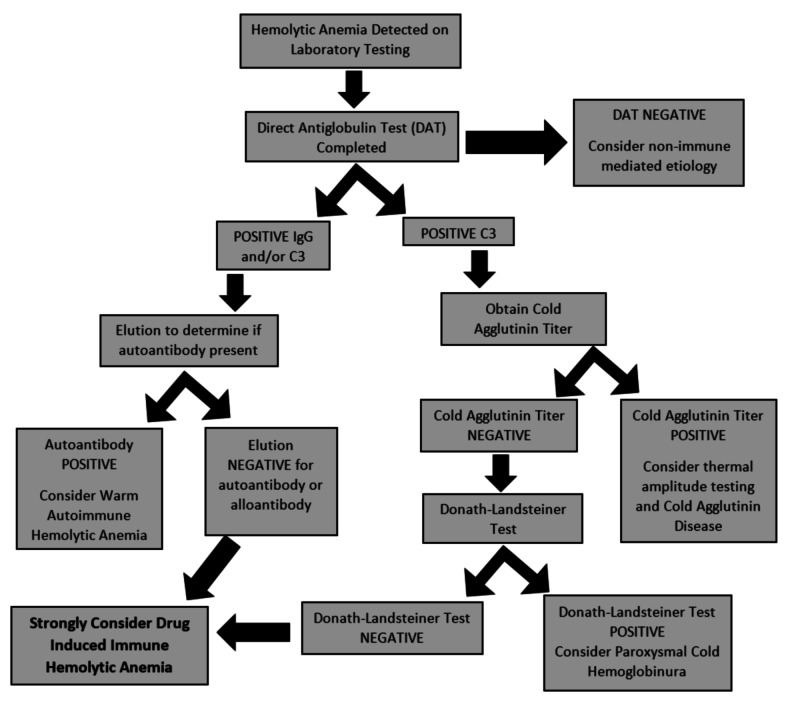
Flowsheet for drug-induced immune hemolytic anemia workup

As in our patient’s case, a DAT with a positive C3 and negative IgG requires further investigation with a cold agglutinin titer to rule out possible cold agglutinin disease [2,7,9]. If cold agglutinin titer is negative, the final step is the completion of the Donath-Landsteiner test. A positive Donath-Landsteiner test demonstrates in vitro hemolysis when temperature of the sample is changed from 4°C to 32°C, but no hemolysis when temperature remains constant at either 4°C or 32°C [7]. The Donath-Landsteiner test is positive in paroxysmal cold hemoglobinuria but negative in DIIHA (Figure 1) [7].

Primary management of DIIHA consists of removing the offending agent [1,3-5,7]. The patient should be supported with RBC transfusions as needed [4,5]. Despite anemia, DIIHA patients are often hypercoaguable and the use of thromboprophylaxis should be strongly considered [4]. Steroids have no proven benefit in DIIHA, but are often used in refractory cases [3,6-8]. If utilized, steroid duration should range from one to three weeks and intravenous steroids should be reserved for those with severe hemolysis [4,8]. When drug-independent autoantibodies are involved and cessation of the offending drug provides no response, intravenous immunoglobulins (IVIG) can be considered as can immunosuppressants, such as cyclophosphamide and azathioprine [3,8]. In rare cases, plasmaphareis may be a therapeutic option, particularly for patients in renal failure [3]. In the majority of cases, clinical and hematological improvement is seen within one to two weeks after cessation of the inciting drug [4,6,8]. All patients should be counseled on future avoidance of the inciting drug for their lifetime and their allergy list should be updated [3,7]. This avoidance should be extended to the drug class involved as cross reactivity is possible [3,7].

## Conclusions

In the presence of new-onset hemolytic anemia and recent antibiotic exposure, DIIHA should remain high on the differential diagnosis. DIIHA is often difficult to differentiate from other autoimmune anemias, a thorough history and serological workup should be obtained. Early identification of DIIHA is crucial as it can be fatal. Removal of the inciting drug is curative in the majority of cases. 

## References

[1] Garratty G (2009). Drug-induced immune hemolytic anemia. Hematology Am Soc Hematol Educ Program.

[2] Mayer B, Bartolmäs T, Yürek S, Salama Salama, A A (2015). Variability of findings in drug-induced immune haemolytic anaemia: experience over 20 years in a single centre. Transfus Med Hemother.

[3] Leicht HB, Weinig E, Mayer B, Viebahn J, Geier A, Rau M (2018). Ceftriaxone-induced hemolytic anemia with severe renal failure: a case report and review of literature. BMC Pharmacol Toxicol.

[4] Hill QA, Stamps R, Massey E, Grainger JD, Provan D, Hill A (2017). Guidelines on the management of drug-induced immune and secondary autoimmune, haemolytic anemia. Br J Haematol.

[5] Garratty G (2020). Immune hemolytic anemia associated with drug therapy. Blood Rev.

[6] Pierce A, Nester T (2011). Pathology consultation on drug-induced hemolytic anemia. Am J Clin Pathol.

[7] Nagao B, Yuan S, Lu Q (2011). Drug-induced immune hemolytic anemia and thrombocytopenia. Transfus Med.

[8] Karunathilaka HGCS, Chandrasiri DP, Ranasinghe P, Ratnamalala V, Fernando AHN (2020). Co-Amoxiclav induced immune haemolytic anaemia: a case report. Case Rep Hematol.

[9] Arndt PA (2014). Drug-induced immune hemolytic anemia: the last 30 years of changes. Immunohematology.

[10] Chen F, Zhan Z (2014). Severe drug-induced immune hemolytic anemia due to ceftazidine. Blood Transfus.

[11] Garratty G (2010). Drug-induced immune hemolytic anemia. Clin Adv Hematol Oncol.

[12] Liebman HA, Weitz Weitz, IC IC (2017). Autoimmune hemolytic anemia. Med Clin N Am.

